# Electro-Therapeutics at Liverpool

**Published:** 1924-12

**Authors:** 


### ELECTRO-THERAPEUTICS AT LIVERPOOL.

At the Royal Southern Hospital, Liverpool, the Earl of Derby has formally opened the new Electro-therapeutic and Massage Department, the buildings of which have been provided by public subscription and the equipment by Sir Alexander and Lady Bicket. In so doing he was continuing the good work of his family, for it was his grandfather who laid the foundation-stone of the hospital in 1867. The new department is furnished with whirlpool, aerated, paraffin-wax and other baths, where over one hundred patients daily are already being treated for paralysis, rheumatic joints, fractures, deformities and all kinds of nervous disorders. This is the only one of the Liverpool hospitals where students can be trained for the examinations of the Chartered Society of Massage and Medical Gymnastics, and it has been a famous training school for nurses since the 'seventies. Recently State Examinations have been instituted.

### Comfort for Nurses.

Great benefit to the nurses is provided by the Nurses' Holiday and Rest Fund, which enables the Committee to provide for the comfort, recreation, and—not least important—education of the nurses. The large private nursing staff has lately had to be curtailed, owing to lack of accommodation caused by the extension of the work together with the shortening of working hours and the consequent enlargement of the hospital nursing staff. Lord Derby presented medals, book prizes and certificates to the nurses successful in the year's examinations, and before leaving promised a gift of £200 for the new department.

### Gravesend Hospital Ladies' Association.

The annual meeting of the Gravesend Hospital Ladies' Association was held recently at the hospital, Mrs. R. H. Foa, the president, presiding. The Association is not purely local, but embraces practically the entire district served by the hospital, and many country members were present. All kinds of useful garments have been made during the past year, whilst a further grant of £30 has been made for the purchase of comfortable chairs for convalescent patients. Again, a grant of £15 was made towards a new typewriter for the hospital secretary's office and a donation of £100 voted to the general funds. The meeting also agreed to try to introduce a scheme in all schools in the district whereby a farthing fund might be commenced with the ultimate view of raising five hundred guineas to endow a schoolchildren's cot. It was reported that a Junior Guild has been commenced in connection with the Association whereby girls and boys not eligible to membership of the Association might become members of the Junior Guild by payment of a subscription of 1s. per annum. Already twenty-five members have joined the Guild. The work done by the Ladies' Association is of the utmost value to the hospital.

